# Babesiosis Surveillance — Wisconsin, 2001–2015

**DOI:** 10.15585/mmwr.mm6626a2

**Published:** 2017-07-07

**Authors:** Elizabeth Stein, Lina I Elbadawi, James Kazmierczak, Jeffrey P. Davis

**Affiliations:** ^1^University of Wisconsin-Madison School of Medicine and Public Health, Preventive Medicine Department; ^2^Career Epidemiology Field Officer, Office of Public Health Preparedness and Response, CDC; ^3^Bureau of Communicable Diseases, Wisconsin Division of Public Health.

Babesiosis is an emerging zoonotic disease caused primarily by *Babesia microti*, an intraerythocytic protozoan. *Babesia microti,* like the causal agents for Lyme disease and anaplasmosis, is endemic to the northeastern and upper midwestern United States where it is usually transmitted by the blacklegged tick, *Ixodes scapularis*. Although babesiosis is usually a mild to moderate illness, older or immunocompromised persons can develop a serious malaria-like illness that can be fatal without prompt treatment. The most common initial clinical signs and symptoms of babesiosis (fever, fatigue, chills, and diaphoresis) are nonspecific and present diagnostic challenges that can contribute to delays in diagnosis and effective treatment with atovaquone and azithromycin (*1*). Results of one study revealed a mean delay of 12–14 days from symptom onset to treatment (*2*). Knowledge of the incidence and geographic distribution of babesiosis can raise the index of clinical suspicion and facilitate more prompt diagnosis and lifesaving treatment (*1*). The first known case of babesiosis in Wisconsin was detected in 1985 (*3*), and babesiosis became officially reportable in the state in 2001. Wisconsin babesiosis surveillance data for 2001–2015 were analyzed in 3-year intervals to compare demographic, epidemiologic, and laboratory features among patients with cases of reported babesiosis. To determine possible reasons for an increase in reported *Babesia* infection, trends in electronic laboratory reporting and diagnosis by polymerase chain reaction testing (PCR) were examined. Between the first and last 3-year analysis intervals, there was a 26-fold increase in the incidence of confirmed babesiosis, in addition to geographic expansion. These trends might be generalizable to other states with endemic disease, similar suburbanization and forest fragmentation patterns, and warming average temperatures (*4*). Accurate surveillance in states where babesiosis is endemic is necessary to estimate the increasing burden of babesiosis and other tickborne diseases and to develop appropriate public health interventions for prevention and practice.

White-tailed deer are the primary hosts for adult blacklegged ticks, and white-footed mice and other small mammals are reservoirs of *B. microti*. Most human cases of babesiosis result from tick bites that occur during the spring and summer months, but blood transfusion–related transmission and perinatal transmission have also been reported (*1**–**3*,*5*). Blacklegged ticks were first recognized in Wisconsin in 1968, and during the subsequent decade, their range expanded rapidly, particularly in northwestern Wisconsin (*6*). Surveys of blacklegged ticks on hunter-harvested deer conducted since 1979 have demonstrated larger numbers of the blacklegged tick population and expansion in geographic range toward northeastern and southeastern Wisconsin (*6*,*7*). The concurrent geographic expansion of blacklegged ticks in Wisconsin during recent decades, coupled with observed increases in reported incidence of other tickborne diseases such as Lyme disease and human anaplasmosis in these regions, highlights the need for accurate surveillance for other serious tickborne diseases, including babesiosis (*8*). Predictive modeling of spatial and temporal trends in tickborne disease in neighboring Minnesota suggests that babesiosis will continue to increase under conditions of warming climate and continued forest fragmentation (*4*). 

In 2001, the Wisconsin Department of Health Services, Division of Public Health defined a confirmed case of babesiosis as the occurrence of fever, anemia, or thrombocytopenia in a patient with confirmatory laboratory findings (i.e., identification of either intraerythrocytic *Babesia* organisms by blood smear or a fourfold increase or greater in *B. microti* immunoglobulin G [IgG] antibody titers). A probable case was defined as the occurrence of fever, anemia, or thrombocytopenia in a patient with supportive positive tests (*B. microti* indirect fluorescent antibody total Ig or IgG antibody titer of ≥1:256 or positive *B. microti* PCR assay). In 2007, the Division of Public Health expanded the confirmed case definition to include a positive PCR result as confirmatory laboratory evidence, which is consistent with the current Council of State and Territorial Epidemiology babesiosis case definition.* For all reported cases, local health departments interviewed health care providers and patients to assess tick exposure and to document the county of exposure and ascertain the possibility of transfusion-associated transmission.

In 2007, the Wisconsin Electronic Disease Surveillance System (WEDSS) was implemented by the Division of Public Health, and electronic laboratory reporting of babesiosis became possible. During the first 3 years of WEDSS implementation, only 17% of confirmed babesiosis cases were initially reported electronically. However, since 2013, approximately 80% of Wisconsin clinical laboratories use electronic laboratory reporting. All cases with either direct or electronic reporting were included in the analysis. Geographic distribution of reported cases by county of residence was compared during five consecutive 3-year intervals to examine geographic expansion of reported babesiosis cases. Annual incidence rates for county and state were calculated using mid-year population estimates provided by the Wisconsin Division of Public Health, Office of Health Informatics. Mean annual incidence was then calculated for successive 3-year intervals.

During 2001–2015, a total of 430 babesiosis cases were reported to the Division of Public Health, including 294 (68%) confirmed and 136 (32%) probable cases. Among confirmed cases, 189 (64%) occurred in males and 199 (68%) in persons aged >60 years (median age = 66 years; range = 10–100 years). Onset of illness occurred during April–October in 283 (96%) reported confirmed cases. Among 242 (82%) patients with confirmed babesiosis for whom sufficient information was available, 158 (65%) were hospitalized. Three deaths occurred, one in a woman aged 88 years, and two in men aged 64 and 72 years; information on comorbid conditions was unavailable. Three confirmed cases of transfusion-associated transmission were detected in 2008 and one in 2011, before implementation of routine screening for babesiosis by Wisconsin blood banks in 2016. Among probable babesiosis cases, 82 (60%) patients were male, 51 (38%) were aged >60 years (median age = 55 years; range = 6–93 years) and 120 (88%) had illness onset during April–October. Among 108 (79%) patients with probable babesiosis for whom information is available, 26 (24%) were hospitalized and none died. The proportion of all cases reported electronically increased to 51% during 2010–2012 and 67% during 2013–2015, compared with 2007–2009 (Figure 1).

**FIGURE 1:**
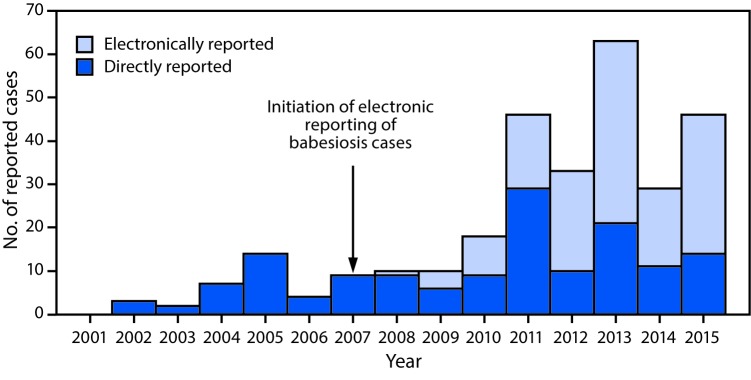
Total confirmed babesiosis case counts (N = 294) initially reported directly and electronically through the Wisconsin Electronic Disease Surveillance System (WEDSS),* Electronic Laboratory Report (ELR) — Wisconsin, 2001–2015 *The WEDSS system records each case report’s first contact source. For example, if a health department or provider notified the Department of Public Health of a case of babesiosis and an electronic report followed, the source would not be categorized as ELR.

From 2001 to 2015 the annual incidence of confirmed babesiosis cases increased during each successive analyzed 3-year interval (Table). During 2001–2003, the mean annual incidence was 0.03 cases per 100,000 Wisconsin residents. During the following 3 years (2004–2006), there was a 400% increase in mean annual incidence to 0.15, followed by a slight (13%) increase to 0.17 during 2007–2009. During 2010–2012, incidence increased sharply, to 0.57, representing a 235% increase compared with the preceding 3 years. During 2013–2015, the mean annual confirmed babesiosis incidence was 0.80 cases per 100,000 Wisconsin residents, representing an overall 26-fold increase compared with 2001–2003.

**TABLE:** Number and incidence of reported confirmed babesiosis cases by 3-year interval and percentage confirmed using polymerase chain reaction (PCR) — Wisconsin, 2001–2015

Years	Confirmed cases of babesiosis		Cases with positive PCR results available*
	No.	Mean annual incidence^†^	No. (% of total cases)
2001–2003	5	0.03	—
2004–2006	25	0.15	3 (38)
2007–2009	29	0.17	8 (28)
2010–2012	97	0.57	53 (77)
2013–2015	138	0.80	95 (86)
**Total 2001–2015**	**294**	**0.34**	**159 (74)**

* Not all reports had laboratory test information available; percentages represent the portions that were positive by PCR among cases with laboratory information available. PCR-positivity was accepted as a criterion for case confirmation in 2007.

**^†^** Per 100,000 state residents.

During 2001–2015, the county of residence was known for all 294 confirmed cases; 50 (69%) of Wisconsin’s 72 counties were represented. The county of likely acquisition was recorded for 163 (56%) confirmed cases, representing 36 counties and one other state (Massachusetts). Among these 163 confirmed cases, the patient’s county of residence and the county of likely tick exposure were the same for 137 cases (84%), representing 33 Wisconsin counties (13 in the northwestern region; nine in the northeastern region, and 11 in the southern region). During 2001–2005, 20 counties (28% of all Wisconsin counties) reported at least one confirmed babesiosis case among residents. The number of counties reporting more than one confirmed babesiosis case among residents increased to 30 during 2006–2010 and to 46 during 2011–2015, representing 42% and 64% of all counties in the state, respectively (Figure 2). This expansion in the geographic range of reported babesiosis cases primarily involved counties to the east and south of the area where babesiosis initially emerged.

**FIGURE 2:**
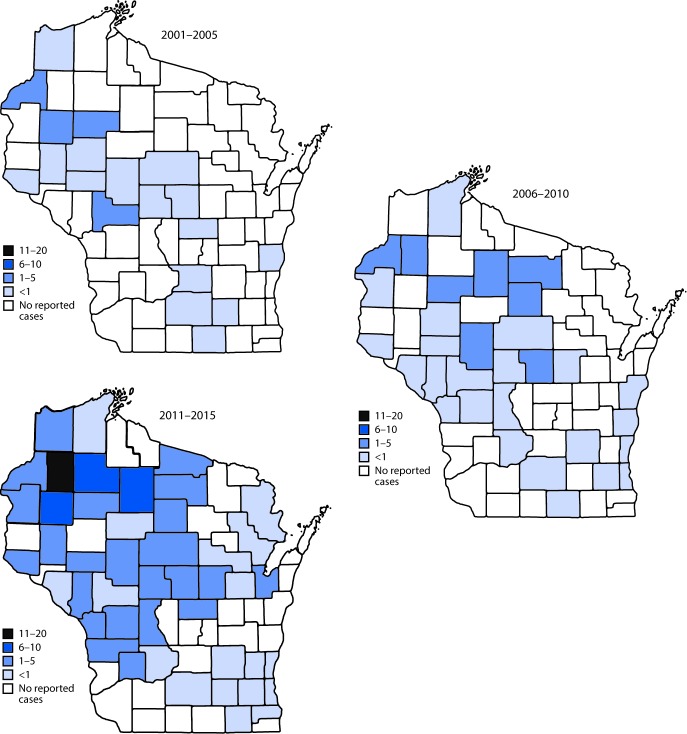
Geographic distribution of confirmed cases of babesiosis per 100,000 residents by county of residence — Wisconsin, 2001–2005, 2006–2010, and 2011–2015 * Twenty counties (28% of all Wisconsin counties) reported at least one confirmed babesiosis case during 2001–2005. During 2006–2010, the number of counties reporting more than one case increased to 30. During 2011–2015, the number of counties reporting more than one confirmed case increased to 46.

## Discussion

The reported incidence of confirmed babesiosis in Wisconsin increased 26-fold from 2001 to 2015, with the greatest increase occurring from 2007–2009 to 2010–2012. In addition to progressive increases in incidence of reported babesiosis, the county of residence of confirmed cases during 2005–2009 and 2011–2015 demonstrated geographic spread to the east and south. Although this substantial increase in reported cases probably reflects an actual increase in incidence, improvements in babesiosis detection and surveillance and increased awareness and diagnoses likely also contributed to this increase.

During 2001–2015 two major changes in babesiosis surveillance occurred that might have affected reported babesiosis incidence rates. The first was expansion of the definition of confirmatory laboratory evidence for babesiosis to include PCR and the second was initiation of automatically generated electronic laboratory reports. Before 2007, peripheral blood smear exam was most frequently used to provide confirmatory laboratory evidence. Blood smear exam has a substantially lower sensitivity of detection of parasites (100–500 parasites/*μ*L blood) than does PCR, which can be positive at concentrations as low as one to three parasites per *μ*L of blood. The inclusion of the more sensitive PCR assay as a confirmatory laboratory criterion, combined with increased use of these tests by providers, likely contributed to an increase in babesiosis diagnoses (*9*).

Improved surveillance also affects reported incidence rates. Before 2010, surveillance for babesiosis relied on manual reporting involving phoned, mailed, or faxed reports from health care providers and laboratories to local health departments or to the Division of Public Health, and these practices might have resulted in underreporting of babesiosis. The marked increase in reported annual incidence rates from 0.17 cases per 100,000 Wisconsin residents during 2007–2009 to 0.57 during 2010–2012 suggests that the shift to automatically generated electronic laboratory reports in 2007 resulted in substantially more confirmed cases being reported.

Despite the observed trend toward routine use of PCR and electronic laboratory reporting, corroborating data from tick surveillance and surveillance of other tickborne diseases suggest a simultaneous actual increase in occurrence of babesiosis in Wisconsin. Documentation of blacklegged tick population expansion to southeastern and northeastern regions of the state suggests that babesiosis has spread to areas that had no previous reports of babesiosis. Also aligning with expanding tick population observations are Division of Public Health anaplasmosis and Lyme disease surveillance data that demonstrate parallel increases in reported incidence in northwestern and central Wisconsin and disease spread toward the southeast and northeast. Because of the extent of improvement in surveillance and diagnostic sensitivity that occurred during 2001–2015, it is difficult to assess the true magnitude of the increase in reported babesiosis incidence during this time. With improved reporting mechanisms and a consistent use of updated case definitions, the accuracy of analyses of trends in reported babesiosis is likely to increase.

The findings in this report are subject to at least two limitations. First, an unknown portion of all babesiosis cases are reported. As noted, more underreporting likely occurred before implementation of automatic reporting via electronic health records. Even after the adoption of the electronic reporting system in 2007, underreporting could have occurred because of the reliance on direct reporting by laboratories not participating in electronic laboratory reporting (estimated to be approximately 20% in 2010) and diagnosis of some cases by blood smear (i.e., not sent to electronic reporting facilities). Second, the geographic distribution of babesiosis cases was estimated using county of residence because county of acquisition could be determined for only 56% of confirmed cases. This could result in underestimation of the cases from more forested and rural counties where Wisconsin residents travel for vacation, while overestimating cases from urban counties where travelers later receive a babesiosis diagnosis.

Increases in the prevalence of tickborne illnesses across the United States are likely, given concurrent evidence of blacklegged tick population growth and geographic expansion, a change that might be attributable to changing weather patterns and increasing forest fragmentation (*10*). Ongoing monitoring of babesiosis incidence using improved surveillance data can help to quantify the burden of disease, prioritize prevention efforts, and raise awareness among health care providers, to ensure timely and correct diagnosis and treatment.

**Summary**

**What is already known about this topic?**

Babesiosis is an emerging tickborne disease endemic to the northeastern United States and the upper Midwest. Many infected persons are asymptomatic but the disease can be life-threatening, especially among older and immunocompromised persons. Prompt diagnosis and treatment in patients with severe infection can prevent serious complications and death.

**What is added by this report?**

Analysis of Wisconsin babesiosis surveillance data during 2001–2015 indicates expansion of the geographic range and increased incidence. Routine use of polymerase chain reaction testing and automatic electronic laboratory reporting likely contributed to the increased reported incidence of confirmed babesiosis in Wisconsin; however, evidence of blacklegged tick expansion suggests an actual increase in infection rates.

**What are the implications for public health practice?**

Babesiosis cases in Wisconsin are increasing in number and geographic range. These trends might be occurring in other states with endemic disease, similar suburbanization and forest fragmentation patterns, and warming average temperatures. Accurate surveillance in states where babesiosis is endemic is necessary to estimate the increasing burden of babesiosis and other tickborne diseases and develop appropriate public health interventions for prevention and practice.

## Footnotes

* http://c.ymcdn.com/sites/www.cste.org/resource/resmgr/PS/10-ID-27.pdf.
